# Heat Stress Influences Immunity Through *DUSP1* and *HSPA5* Mediated Antigen Presentation in Chickens

**DOI:** 10.3390/ani15081141

**Published:** 2025-04-16

**Authors:** Xiaomeng Wang, Xiaohuan Chao, Wenwu Zhang, Xiquan Zhang, Jiongwen Wu, Chutian Ye, Xuerong Ma, Zhexia Fan, Manqing Liu, Cheng Fang, Qingbin Luo

**Affiliations:** 1 State Key Laboratory of Livestock and Poultry Breeding, South China Agricultural University, Guangzhou 510642, Chinaxqzhang@scau.edu.cn (X.Z.);; 2 College of Animal Science, South China Agricultural University, Guangzhou 510642, China

**Keywords:** chicken, immune response, mRNA-seq, *DUSP1*, *HSPA5*

## Abstract

The objective of this study was to elucidate the immune system response to heat stress in chickens. In this study, mRNA-seq was conducted on the spleen and bursa of experimental chickens, six differentially expressed genes associated with immunity were present in the spleen following immunization. Following exposure to heat stress, 15 differentially expressed genes related to immune and heat shock proteins were identified. Furthermore, the expression levels of *DUSP1* and *HSPA5* were significantly lower in the non-stressed group. With regard to the mechanism, overexpression of *DUSP1* or *HSPA5* resulted in no significant difference in *MHC-I*, *MHC-II*, and *CD80* mRNA expression. However, following stimulation with LPS, mRNA expression of *MHC-II*, *CD80*, *CD86*, *CD1C*, *IL1B*, and *TLR4* was significantly increased. Furthermore, the enhancement was observed to occur at an earlier stage than when LPS was stimulated alone, thereby facilitating the recognition of LPS by HD11. Following the inhibition of DUSP1 or HSPA5 and the stimulation of LPS, no significant alterations were detected. However, CD1C expression was notably diminished. In conclusion, *DUSP1* and *HSPA5* have been demonstrated to play important roles in immunity to heat stress by affecting antigen presentation. The present study provides a theoretical basis for the regulation mechanism of disease resistance in poultry.

## Simple Summary

High temperature is a major stressor that has been shown to impair poultry health and performance through multiple pathways, including metabolism and immunity. This has significant negative consequences for the poultry industry. In this study, mRNA-seq was used to identify differentially expressed genes (DEGs) in the spleen and bursae of Fabricius of chickens during heat stress. The DEGs included *IGSF11*, *ALDOB*, *APOB*, *GZMA*, *DUSP1*, and *HSPA5*. Subsequent cell function analysis confirmed that the over-expression of *DUSP1* and *HSPA5* enhanced the capacity of HD11 cells to recognize and bind LPS while concomitantly reducing the time required for LPS presentation. This study provides a theoretical foundation for further research on the impact of heat stress on the immune system.

## 1. Introduction

Under high temperatures in summer, poultry would experience a host of health problems, such as reduced immunity and disease resistance, lower food intake, slow growth and development, abnormal endocrine functions, and increased mortality [1,2,3,4,5]. These issues seriously hamper intensive poultry production, leading to economic losses. High temperatures usually cause heat stress, which could influence chicken productive performance through reduced immunity. Effect of chronic heat stress on some physiological and immunological parameters in different breeds of broilers [6,7,8].

It has been demonstrated that acute heat stress results in the inhibition of pituitary and thyroid components within the pituitary–thyroid–peripheral tissue axis (PTTA). Furthermore, lipopolysaccharides (LPSs) have been shown to exert additional inhibitory effects on all components of the PTTA [9]. As a highly conserved protein family, the heat shock proteins (HSPs) are expressed under high temperatures or other environmental stresses, such as hypoxia, ultraviolet radiation, cold, viral infection, and starvation, and bind to non-protein molecules with exposed hydrophobic residues [10,11,12,13]. Mammalian *HSP60*, *HSP70*, *HSP90*, and *gp96* can form bonds with LPS [14,15,16]. In mammals, other functions of HSPs include inducing antigen-presenting cells (APCs) to secrete cytokines (*IL-1* and *IL-6*), promoting dendritic cell maturation, and increasing *MHC-I* and *MHC-II* expression [17,18,19]. Extracellular HSPs can also promote cross-presentation of HSP-bound peptide antigens to *MHC-I* molecules in dendritic cells, activating antigen-specific cytotoxic T lymphocytes [20]. There are differences in chickens and mammals on immune genes, and it is important to conduct research to better understand these differences.

The pecteneal hyalocytes can recognize antigens but cannot present antigens in chickens, which are considered to be monocyte/macrophage systems, and the function is not yet fully understood in the immune response [21]. The immune response of the HD11 (a macrophage cell line) to LPS in the heat-stress environment (up-regulation of *CCL4*, *CCL5*, *IL1B*, *IL8,* and *iNOS*) was higher than in thermoneutral conditions [22]. Expression for the immune-related genes *CCL4*, *CCL5*, *CD40*, *GM-CSF*, *IFN-γ*, *IL-10*, *IL-12b*, *IL-1b*, *IL-6*, *IL-8*, and *iNOS* was highly induced in response to LPS in the chicken-bone-marrow-derived dendritic cells [23]. Microarray analysis on chicken DNA has revealed the transcriptome of E. coli-infected chicken HD11, and researchers speculate that chicken HSP70 is involved in immune-related pathways [24]. The involvement of avian HSPs in immune response was further substantiated by the observation that the inhibition of intracellular *HSP90* expression led to a notable reduction in IFN-γ expression following the immunostimulation of chicken HD11 cells with CpG oligodeoxynucleotides (CpG-OND). Conversely, *HSP90*-bound CpG-OND increased *IFN-γ*, *IL-6*, and *MIP-3α* expression, as well as nitrogen oxide (NO) levels [24].

HSPs have been demonstrated to influence immune response. However, research into the function of *HSPA5* in antigen presentation remains limited. In this study, mRNA-seq was employed to identify genes associated with heat stress, and the impact of *DUSP1* and *HSPA5* on antigen presentation was validated through cell function analysis. This will facilitate further investigation into the role of antigen presentation in immune function during heat stress.

## 2. Materials and Methods

### 2.1. Experimental Animal Management

Twelve 28-day-old SPF chickens (Xinxing Dahuanong Poultry and Egg Co., Ltd, Guangdong, China) were inoculated with Newcastle disease vaccine (NDV, Live, Strain Clone 30, Harbin, China, only as antigen) and served as an experimental group, and six 28-day-old SPF chickens were not inoculated with Newcastle disease vaccine and served as a control group. Antibody levels were detected after 7 d of rearing. The experimental chickens were divided into three groups: control group (not been vaccinated against Newcastle disease; *n* = 6; 24 ± 1 °C for 3 h), vaccination group (vaccinated against Newcastle disease but had non-stressed; *n* = 6; 24 ± 1 °C for 3 h), and heat treatment vaccination group (vaccinated against Newcastle disease but had experienced stressed; *n* = 6; 36 ± 1 °C for 3 h). They were stunned with a stun gun and promptly exsanguinated through the jugular vein at 35 days old. Subsequently, three chicks were selected from each group, and the spleen and bursa of Fabricius tissue of these chicks were harvested for mRNA-seq. Blood samples were collected and stored at −20 °C. Tissue samples were stored in liquid nitrogen. The experimental scheme was approved by the Animal Care Committee of South China Agricultural University.

### 2.2. Antibody Level Determination and Measurements of Heat-Resistance Traits

Hemagglutination inhibition (HI) antibody levels were measured according to previously reported [25]. Corticosterone was detected using an enzyme-linked immunosorbent assay (ELISA) kit (Guangzhou Darui Antibody CO., LTD, Guangzhou, China) as per the manufacturer’s instructions. The H/L value was counted by light microscope, and the expression of CD3^+^, CD4^+^, and CD8^+^ T cells was analyzed using flow cytometry.

### 2.3. mRNA-Seq of Spleen and Bursa of Fabricius Tissues

**Figure A1:**
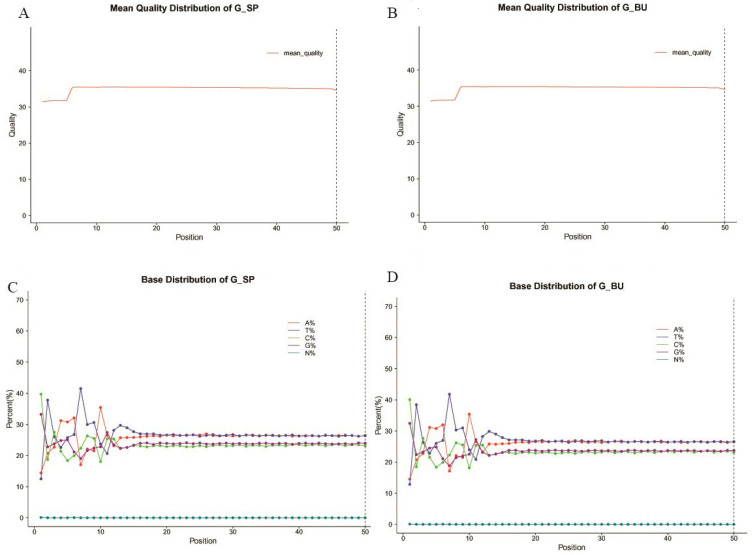
Data quality of sequencing samples. (**A**,**B**) Base mass fraction of all samples sequenced reads. (**C**,**D**) Base composition of the sequenced sample.

The spleen and bursae of Fabricius from the control group, the vaccination group, and the heat treatment vaccination group were subjected to mRNA-seq (Illumina HiSeq 2500, SE50, 5×). Each treatment had three biological replicates, totaling 18 samples. All samples’ base mass fractions of sequencing reads were above Q30 (99.9% recognition rate of bases) (Figure A1A,B). In the base composition of each sequencing sample, C and G curves mostly overlapped, as did T and A curves. The sequencing process was stable and could be visualized as a horizontal line, indicating balanced base composition in each sequencing sample (Figure A1C,D). Sequenced reads were trimmed for adaptor sequence and masked for low-complexity or low-quality sequence, then mapped to *Gallus gallus* (galgal6) whole genome using bowtie 2. RefSeq identifiers of candidate genes were matched to corresponding Ensembl identifiers (Ensembl release 97, www.ensembl.org, 3 October 2019). Differential expression analysis of two conditions/groups was performed using the DEGseq. Genes with an adjusted *p*-value < 0.05 found by DEseq were assigned as differentially expressed. Gene Ontology (GO) enrichment analysis of the DEGs was implemented by the GOseq R packages (Bioconductor 3.18) based on Wallenius non-central hyper-geometric distribution [26], which can adjust for gene length bias in DEGs. We used KOBAS 2.0 software (http://bioinfo.org/kobas, 5 December 2019) to test the statistical enrichment of differential expression genes in KEGG pathways [27].

### 2.4. Cell Culture

The chicken HD11 macrophage cell line (gifted from Prof. Susan Lamont, Department of Animal Science, Iowa State University, Ames, IA, USA) was cultured at 37 °C and 5% CO_2_ in Roswell Park Memorial Institute 1640 medium (Gibco Life Technologies, Carlsbad, CA, USA) supplemented with 10% heat-inactivated fetal calf serum (PAA, Pashing, Austria), 10 mm HEPES, 0.1 mm non-essential amino acids, 2 mm glutamine, 1 mm sodium pyruvate, 100 U/mL penicillin, 100 μg/mL streptomycin, and 5 × 10^−5^ M 2-mercaptoethanol (pH 7.3).

### 2.5. Synthesis of siRNA and LPS Stimulation

A set of four siRNA interfere fragments was synthesized by GenePharma (GenePharma, Suzhou, China) that targeted distinct sites within *HSPA5* and *DUSP1*, along with a positive control targeting glyceraldehyde-3-phosphate dehydrogenase (GAPDH) (sequences are shown in Supplemental Data 2). Cells (1–2 × 10^5^) seeded in Costar 12-well plates (Corning, Palo Alto, CA, USA) were stimulated with 1 μg/mL LPS (E. coli endotoxin 0111: B4; Sigma, St. Louis, MO, USA). Cells were collected for RNA extraction 0, 2, 4, and 6 h post stimulation. To verify the role of *HSPA5* and *DUSP1* in LPS-induced activation, HD11 cells were transfected with an *HSPA5* and *DUSP1* overexpression plasmid or treated with the siRNA interfere fragments targeting *HSPA5* and *DUSP1* for 48 h, then stimulated with LPS 1 μg/mL, and collected 0, 2, 4, and 6 h post stimulation. Triplicate samples were used in each group.

### 2.6. Overexpression Plasmid Construction and Transfection

Chicken cDNA was synthesized from RNA using the iScript cDNA Synthesis Kit (Bio-Rad, Hercules, CA, USA). The chicken *DUSP1* open reading frame (the Hind III digestion site was added to the 5′ end, and the Xba I restriction site was added to the 3′ end) was synthesized by the company (Gene Create, Wuhan, China). The product was cloned into the pcDNA3.1 vector between the Hind III and Xba I restriction sites. The primer pair 5′-ggggGGTCTCtagtgGTTCGTGTGTGACGAGCGG-3′ (forward) and 5′-ccgGGTCTCgtgggACACAACATTCAGAGATGCCAGT-3′ (reverse) was used to amplify the chicken *HSPA5* open reading frame (uppercase in the primer sequences, annealing temperature is 56 °C). The PCR product was cloned into the pSDS vector following the RuyilianTM instructions (Innovative Cellular Therapeutics, Shanghai, China); 0.8 μg plasmid and 3 μL Attractene (Qiagen, Düsseldorf, Germany) were mixed with 60 μL Opti-MEM (Gibco Life Technologies) and incubated for 12 min at room temperature, then transfected into cells.

### 2.7. Reverse Transcription-qPCR (RT-qPCR) Analysis

Total RNA was extracted from cells using TRIzol reagent (Takara Bio Inc., Dalian, China), and first-strand cDNA was synthesized using iScript cDNA Synthesis kit (Bio-Rad). The cDNA samples were quantified by RT-qPCR using the CFX96 system with SsoFast EvaGreen Supermix (Bio-Rad), and reaction conditions were set according to the manufacturer’s recommendations with an annealing temperature of 58–65 °C (40 cycles). The primer sequences are shown in Table A1. *GAPDH* was used as an internal control for RT-qPCR data normalization.

### 2.8. Statistical Analysis

A Student’s *t*-test was employed to ascertain the disparities between the groups. Each group comprised at least three replicates. GraphPad software was used for data analysis (GraphPad Prism 8). Differences were analyzed using the Student’s *t*-test. A value of *p* < 0.05 was considered to be statistically significant.

## 3. Results

### 3.1. Antibodies, Along with Pre and Post-Heat-Stress Changes to Physiological Indices

**Figure 1:**
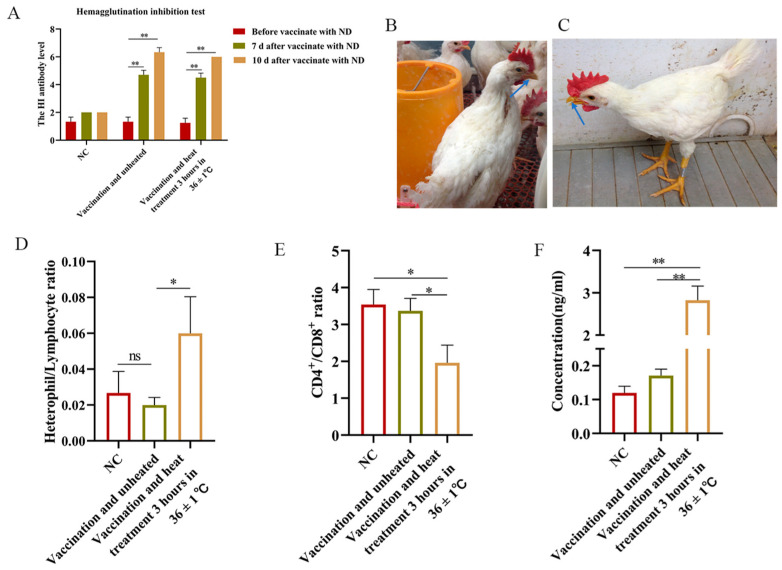
Antibody levels and heat stress detection. (**A**) Detection of antibody levels after NDV injection. (**B**) The chickens in the NC group were normal breathers. (**C**) The chickens in the heat stress (36 ± 1 °C) group showed mouth opening and rapid breathing. (**D**–**F**) The changes in H/L, concentration, and CD4^+^/CD8^+^ were detected after different treatments. Data presented as mean ± SEM. Blue arrow: The state of the chicken's mouth; *: *p* <  0.05; **: *p*  <  0.01, ns: no significance.

Following the administration of Newcastle disease virus, antibody levels in heat-stressed SPF chickens demonstrated a significant increase at 7 and 10 days post-immunization, indicating the persistence of an immune response. Moreover, acute heat stress did not appear to cause antibody changes immediately (Figure 1A). No tachypnoea was observed in the NC group, whereas tachypnoea was observed in the group exposed to heat stress (36 ± 1 °C, Figure 1B,C). We then investigated the changes in physiological parameters in response to stress in different treatment groups. They had significantly higher H/L (*p* < 0.01) and corticosterone (*p* < 0.01) (Figure 1D,F) and significantly lower CD4^+^/CD8^+^ ratio (*p* < 0.01) than non-heat-stressed chickens (Figure 1E), indicating a physiological stress response.

### 3.2. Gene Expression Differences in Spleen and Bursa of Fabricius Tissues from Different Treatments

**Figure 2:**
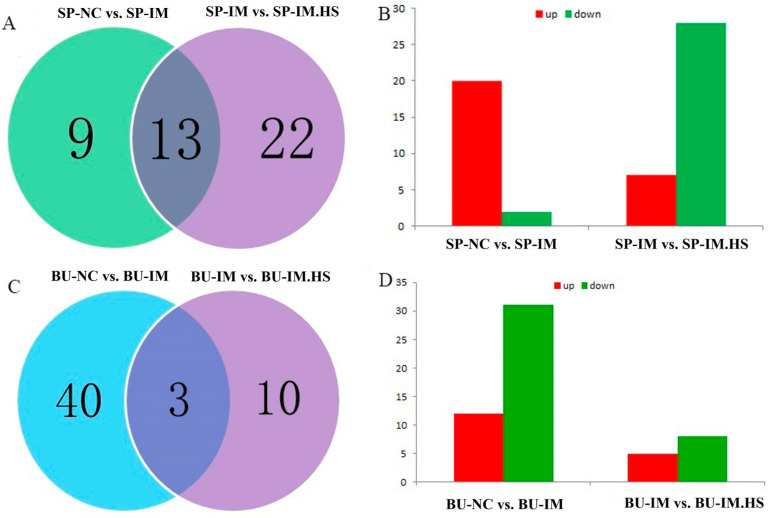
Differentially expressed genes among four contrasts. The spleen-sequencing data were subjected to grouping and comparison. The SP-NC is the control group, SP-IM is the vaccination group, and SP-IM. HS refers to the heat treatment vaccination groups (**A**,**B**). The sequencing data pertaining to the bursa of Fabricius were subjected to grouping and comparison. BU-NC is the control group, BU-IM is the vaccination group, BU-IM.HS refers to the heat treatment vaccination groups (**C**,**D**). (**A**,**C**): DEGs are unique or shared among the two contrasts; (**B**,**D**): DEGs are up or down in the latter of each contrast.

The mRNA-seq analysis yielded a total of 22 differentially expressed genes in Group A, of which 20 were found to be up-regulated and 2 were down-regulated, including *FGB*, *IGSF11*, and *ALDOB* (Figure 2A). Similarly, Group B exhibited a total of 35 differentially expressed genes, encompassing 7 up-regulated genes and 28 down-regulated genes, including *GZMA*, *STAR*, and *DUSP1* (Figure 2B). Group C revealed a total of 43 genes, of which 12 were found to be up-regulated, while 31 were down-regulated, including *MAP2*, *CD69*, and *MYL4* (Figure 2C). Group D exhibited a total of 13 genes, of which 5 were up-regulated and 8 were down-regulated, including *ABCB1LB*, *COL21A1*, and *CD69* (Figure 2D).

### 3.3. GO Enrichment Analysis of Differentially Expressed Genes in the Spleen and Bursa of Fabricius

**Figure 3:**
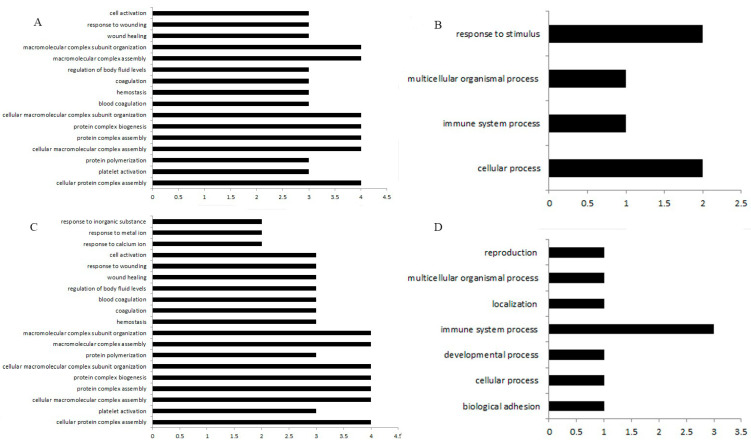
The biological processes of DEGs enrichment in different treatments. (**A**) GO enrichment analysis of DEGs in spleen samples. (**B**) Enriched biological processes included the assembly of cellular protein complexes, platelet activation, etc. (**C**) GO enrichment analysis of DEGs in the bursa of Fabricius samples. (**D**) Enrichment occurred in adhesion and cellular processes.

The GO enrichment analysis found that the differentially expressed genes in the spleen samples of Group A were enriched in the following biological processes: cellular protein complex assembly (4 genes, *p* < 0.05), platelet activation (3 genes, *p* < 0.05), and assembly of cellular macromolecule complexes (4 genes, *p* < 0.05) (Figure 3A). In Group B, enriched biological processes included the assembly of cellular protein complexes (4 genes, *p* < 0.05), platelet activation (3 genes, *p* < 0.05), and protein aggregation (3 genes, *p* < 0.05) (Figure 3B). Differentially expressed genes in the bursa of Fabricius samples of Group C were enriched in the following biological processes: cellular processes (2 genes, *p* < 0.05), multicellular processes (1 gene, *p* < 0.05), and responses to stimuli (2 genes, *p* < 0.05) (Figure 3C). In Group D, enrichment occurred in biological adhesion (1 gene, *p* < 0.05) and cellular processes (1 gene, *p* < 0.05) (Figure 3D).

### 3.4. Differentially Expressed Genes Associated with Immune Responses in the Spleen and Bursa of Fabricius

By analyzing the differentially expressed genes, we found that six differentially expressed genes (including *IGSF11*, *ALDOB*, and *APOB*) were associated with spleen immune response after immunization. Their upregulation is associated with enhanced immunity, suggesting that immunity raises disease resistance in chickens. Additionally, 15 differentially expressed genes (*GZMA*, *DUSP1*, and *HSPA5*) associated with immune response and heat stress were identified after 3 h of heat stimulation (Table 1). The upregulation of *GZMA* can enhance immunity, while the downregulation of *DUSP1* and *HSPA5* is associated with the decrease in immunity, indicating that the disease resistance of chickens is negatively affected by heat stress, and *GZMA* may play a negative feedback role in the decrease in immunity under stress conditions.

In the bursa of Fabricius, *CD69* was the only immune-response-related gene identified after immunization. After 3 h of heat stimulation, three more differentially expressed genes (*COL21A1*, *CD69*, and *HSP90AB1*) associated with immune response were detected (Table 1). Contrasting to results from spleen samples, these data indicated that the bursa of Fabricius is insensitive to heat stress and the vaccine used.

### 3.5. KEGG Pathway Analysis of Differentially Expressed Genes in the Spleen and Bursa of Fabricius

The results of the KEGG pathway analysis showed that the 22 differentially expressed genes in Group A were significantly enriched in 8 pathways (*p* < 0.05), including complement and coagulation cascades, pentose phosphate pathway, and fat digestion and absorption (*p* < 0.05). In Group B, 35 genes were enriched in 7 pathways, such as glutathione metabolism, complement and coagulation cascades, and chemokine-signaling pathways (Table 2). We noted that the pathway of complement and coagulation cascades was found in both groups. These pathways involve proteins mediating immune and inflammatory responses. Thus, KEGG results suggested that the genes in Groups A and B are associated with immune responses. The 43 differentially expressed genes in Group C were not enriched in any pathway, whereas the 13 differentially expressed genes in Group D were significantly enriched in the following 2 pathways, synaptic vesicle cycle and protein digestion and absorption (Table 2). These results further emphasize that the bursa of Fabricius does not respond to heat stress or the selected vaccine.

### 3.6. Ingenuity Pathway Analysis (IPA) Network Formed by Differentially Expressed Genes in the Spleen and Bursa of Fabricius

**Figure 4:**
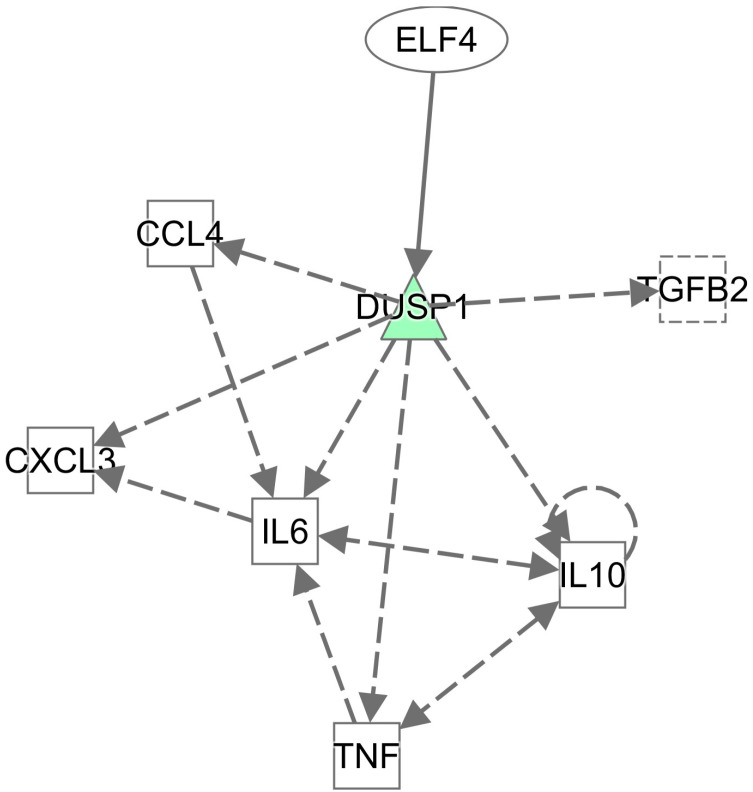
IPA network of differential gene enrichment. The 35 differentially expressed genes in Group B formed significantly interactive gene networks. Colored in green are down-regulated in White Leghorn. Color intensity correlates with the size of the FC, in which the color is darker, and the variance is greater.

The results of the IPA network analysis on Group A’s 22 differentially expressed genes demonstrated that they formed a significantly interactive gene network (Table 3), associated with cellular functions and maintenance, hematopoietic system development and function, as well as cell development. The 35 differentially expressed genes in Group B formed 2 significantly interactive gene networks (Table 3). The first was associated with neurological system development and function, cancer, as well as cardiovascular system development and function. The second was associated with cell motility, along with multiple immunity-related genes, including hematopoietic system development and functions, along with immune cell trafficking (Figure 4). In short, these networks in the spleen have a strong association with immune response. Group C’s 43 differentially expressed genes also formed two significantly interactive gene networks (Table 3). The first was associated with cell migration, along with cellular functions, maintenance, and behavior, whereas the second was associated with cell-to-cell signal transduction and interactions, digestive system development and function, as well as liver development and function. The 13 differentially expressed genes of Group D formed a single significantly interactive gene network (Table 3), associated with cell-to-cell signal transduction and interactions, as well as cell growth, proliferation, and migration. In line with data from our other analyses, these networks in the bursa of Fabricius have no strong association with immune response.

### 3.7. RT-qPCR Analysis of the Differentially Expressed Genes

To verify sequence accuracy, 20 differentially expressed genes were randomly selected from Groups A–D for RT-qPCR verification. The results indicated different fold changes (FC) from the sequencing data but consistent trends in differential expression (Table 4). Thus, the sequencing results appeared to be reliable.

The sequencing and RT-qPCR results all indicated that heat stress significantly decreased *DUSP1* (sequencing: FC = 0.29, *p* < 0.01; RT-qPCR: FC = 0.45, *p* < 0.01) and *HSPA5* (sequencing: FC = 0.52, *p* < 0.01; RT-qPCR: FC = 0.42, *p* < 0.01). Both KEGG and IPA analyses indicated that *DUSP1* is involved in immune-related pathways, and many reports have shown that *HSPA5* is associated with immune response. Thus, our subsequent experiments focused on verifying that *DUSP1* and *HSPA5* differential expression was associated with post-heat-stress immunity.

### 3.8. LPS Stimulates Immune Response in Chicken HD11 Cells

**Figure 5:**
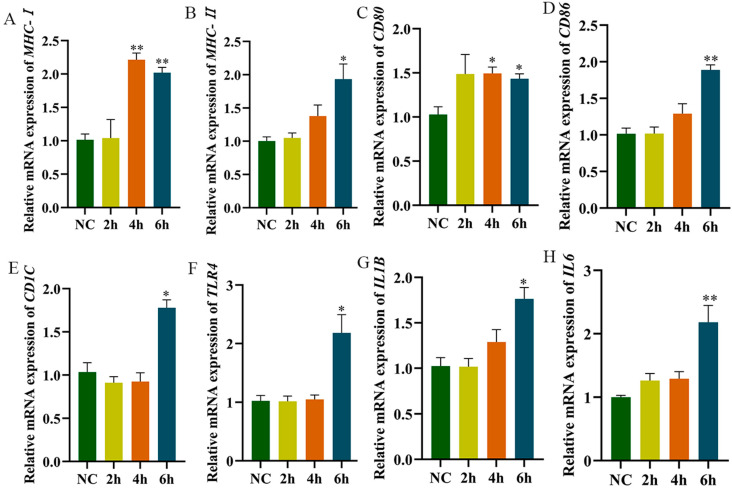
The expression detection of related genes after LPS-stimulated HD11. (**A**–**H**): Cells were collected for RNA extraction after being stimulated for 2 h, 4 h, and 8 h using 1 μg/mL of LPS; RT-qPCR was used to detect the expression level of related genes. NC is the control group, 2 h, 4 h, and 6 h are the stimulation times by LPS. The NC group is without LPS stimulation. Data presented as mean ± SEM, *: *p*< 0.05; **: *p* < 0.01.

The results of the RT-qPCR showed that compared with the control group, mRNA levels of *MHC-I* and *CD80* were very significantly or significantly increased at 4 h post-LPS stimulation. At 6 h post-stimulation, *MHC-II*, *IL1B*, *IL6*, *TLR4*, *CD1C*, *CD80*, and *CD86* mRNA levels were very significantly or significantly increased (Figure 5A–H). Furthermore, *MHC-II* expression on HD11 cell surfaces significantly increased at 4 h after LPS stimulation (Figure 5).

### 3.9. The Effect of DUSP1 on LPS Recognition in HD11

**Figure 6:**
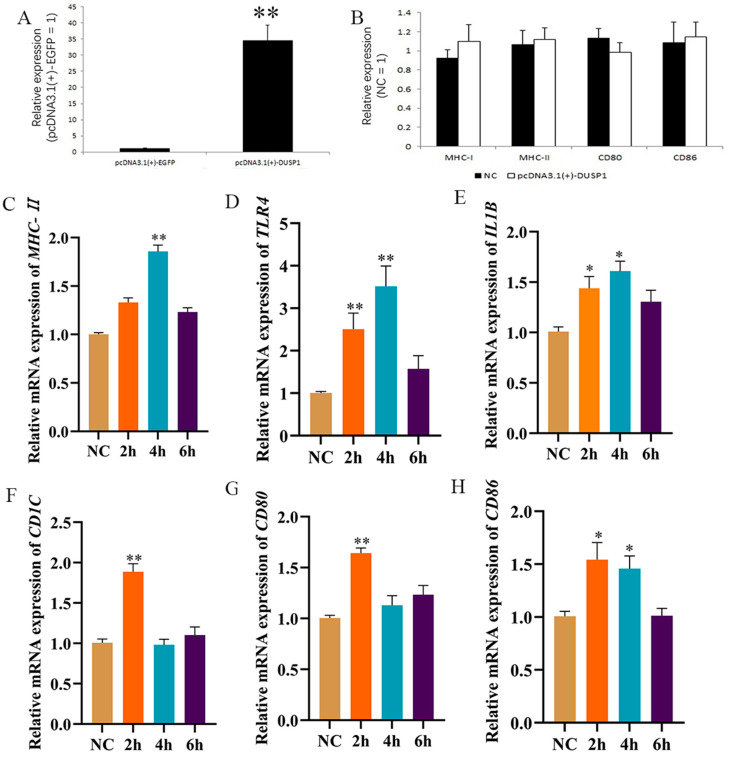
*DUSP1* overexpression accelerates HD11 recognition of LPS and thus allows earlier LPS presentation. (**A**) Verification of *DUSP1* overexpression efficiency. (**B**) RT-qPCR was used to detect antigen-passing-related genes after overexpression of *DUSP1*. (**C**–**H**) Overexpression of *DUSP1* was followed by 1 μg/mL LPS stimulation for different times, followed by RT-qPCR to detect the expression of *MHC-II*, *TLR4*, *IL1B*, *CD1C*, *CD80*, and *CD86*.The NC group was only stimulated with LPS.Data presented as mean ± SEM, *: *p*< 0.05; **: *p* < 0.01.

To ascertain the biological function of *DUSP1*, the pcDNA3.1-*DUSP1* overexpressing vector was constructed (Figure 6A). Then, subsequent to the transduction of pcDNA3.1-*DUSP1* into HD11 cells. RT-qPCR results showed that the mRNA expression of *MHC-I*, *MHC-II*, *CD80*, and *CD86* did not significantly differ before and after *DUSP1* overexpression (Figure 6B). The above results show that intracellular *DUSP1* overexpression does not influence macrophage activation without actual LPS addition. We performed LPS stimulation after overexpression of *DUSP1* in HD11 cells. The RT-qPCR results showed that *TLR4* expression was significantly up-regulated at 2 h after LPS stimulation (Figure 6D). Moreover, a two-fold decrease in duration before *TLR4* up-regulation occurred compared with LPS stimulation alone. We also found that *MHC-II* mRNA levels increased significantly at 4 h after LPS stimulation (Figure 6C), while mRNA levels of IL1B (Figure 6E), *CD1C* (glycolipid antigen-presenting molecule) (Figure 6F), *CD80* (Figure 6G), and *CD86* (Figure 6H) increased significantly at 2 h after LPS stimulation. This timing was earlier than under LPS stimulation only. Together, these data indicated that *DUSP1* overexpression accelerates HD11 recognition of LPS and thus allows earlier LPS recognition, as compared with LPS stimulation alone.

**Figure 7:**
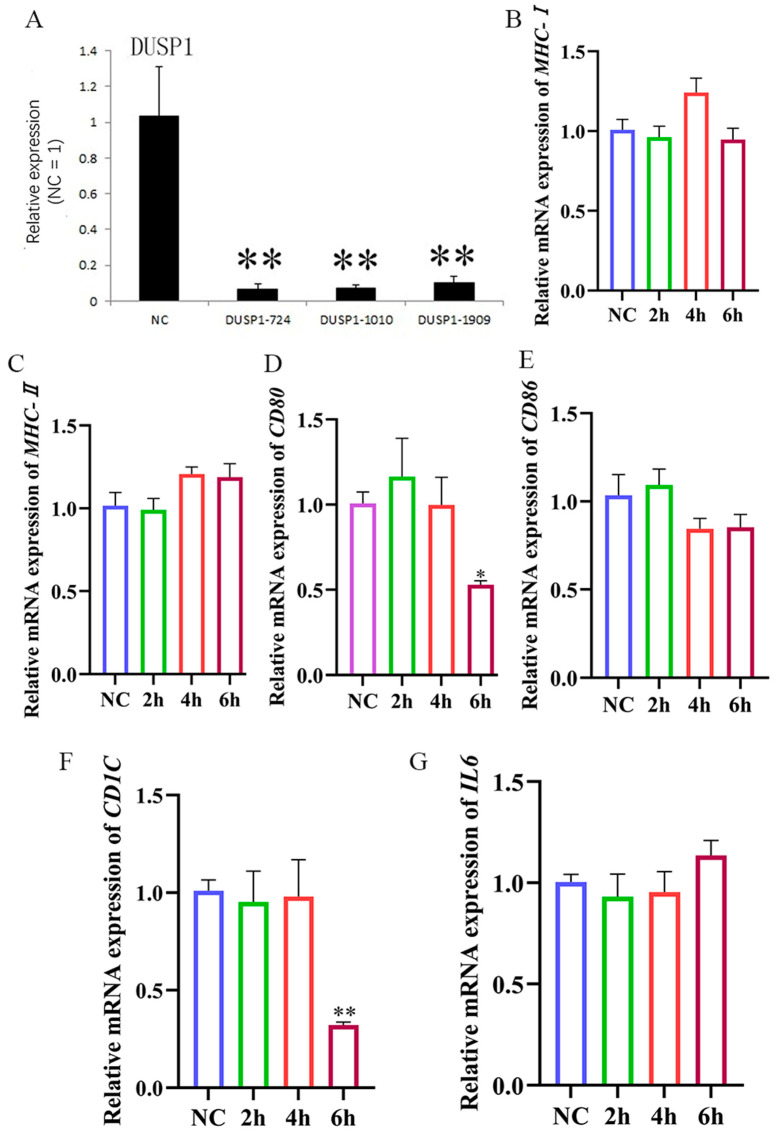
HD11 presentation of LPS is affected by *DUSP1* expression levels. (**A**) Interference effect of *DUSP1* interference fragment. (**B**–**G**): After interfering with *DUSP1*, 1 μg/mL LPS stimulation was given at different times, followed by RT-qPCR to detect the expression of *MHC-I, MHC-II, CD80*, *CD86*, *CD1C*, and *IL6*.The NC group was only stimulated with LPS. Data presented as mean ± SEM, *: *p*< 0.05; **: *p* < 0.01.

The *DUSP1* interfering fragment 724 (*DUSP1*-724) was able to inhibit *DUSP1* expression (Figure 7; Table A2). After treatment with *DUSP1*-724, HD11 cells were stimulated with LPS, and the results within 6 h showed that the levels of *MHC-I* (Figure 7B) and *MHC-II* (Figure 7C) mRNA remained largely unaltered in comparison to the levels observed in the LPS stimulation group. Thus, interfering with *DUSP1* can inhibit *MHC-I* and *MHC-II* expression in LPS-stimulated HD11 cells. We also found that the *CD80* (Figure 7D) and *CD86* (Figure 7E) mRNA expression was inhibited within 6 h post-LPS stimulation, followed by a significant decrease also observed in the mRNA expression of *CD1C* mRNA expression (Figure 7F). In addition, we found that *IL6* expression (Figure 7G) failed to change significantly within 6 h post-LPS stimulation, suggesting a decreased ability of HD11 cells to secrete cytokines upon interference with *DUSP1* expression. In summary, our data suggested that HD11 recognition of LPS is affected by *DUSP1* expression levels. Thus, interfering with *DUSP1* inhibits the ability of HD11 recognition LPS, causing downstream effects in the expression of other immune-related genes.

### 3.10. Effect of HSPA5 on the Ability of HD11 to Recognize LPS

**Figure 8:**
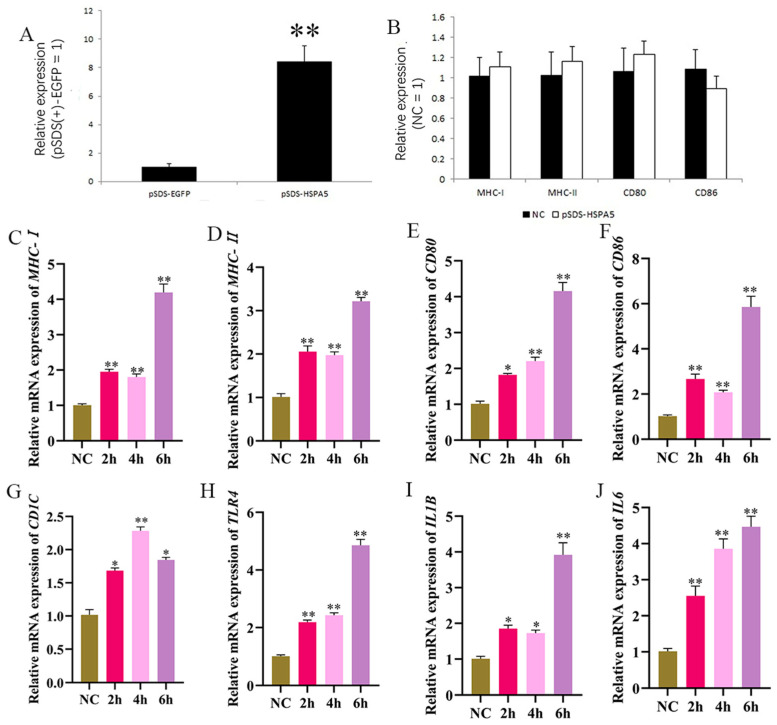
*HSPA5* overexpression accelerates HD11 recognition of LPS and thus allows earlier LPS presentation. (**A**) Verification of *HSPA5* overexpression efficiency. (**B**) Detection of antigen passing-related genes after overexpression of *HSPA5*. (**C**–**J**) Overexpression of *HSPA5* was followed by 1 μg/mL LPS stimulation for different times, followed by RT-qPCR to detect the expression of *MHC-I, MHC-II*, *CD80*, *CD86*, *CD1C*, *TLR4*, *IL1B*, and *IL6*.The NC group was only stimulated with LPS.Data presented as mean ± SEM, *: *p*< 0.05; **: *p* < 0.01.

To ascertain the biological function of *HSPA5*, the pSDS-*HSPA5* overexpressing vector was constructed (Figure 8A). We tested whether changes to intracellular *HSPA5* expression can alter macrophage activation through the RT-qPCR. The mRNA expression level of intracellular *MHC-I*, *MHC-II*, *CD80*, and *CD86* did not change significantly before and after *HSPA5* overexpression (Figure 8B), indicating that without LPS addition, *HSPA5* overexpression does not affect macrophage activation. LPS was added after overexpression. The mRNA levels of *MHC-I*, *MHC-II*, *CD80*, *CD86*, *CD1C*, *IL1B*, *IL6*, and *TLR4* (Figure 8C–J) increased significantly at 2 h after LPS stimulation when *HSPA5* was overexpressed. This increase occurred far earlier than the timing of *MHC-II* expression after LPS stimulation alone. Together, these data combine to demonstrate clearly that *HSPA5* can facilitate and accelerate HD11′s LPS recognition.

**Figure 9:**
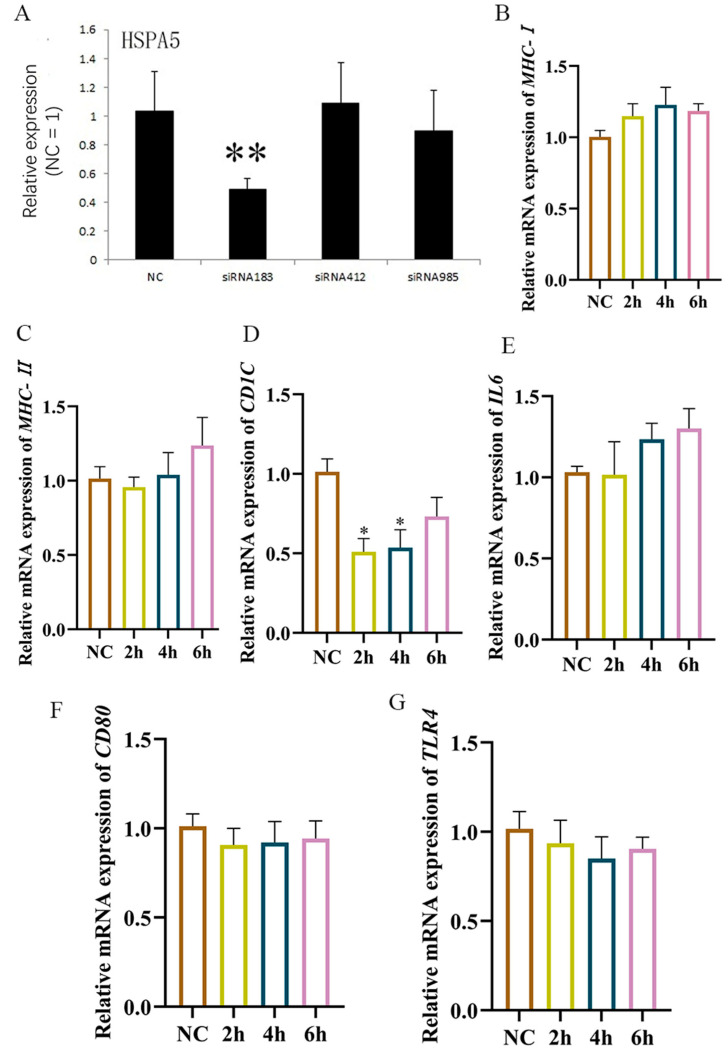
HD11 presentation of LPS is affected by *HSPA5* expression levels. (**A**) Interference effect of *HSPA5* interference fragment; (**B**–**G**): After interfering with *HSPA5*, 1 μg/mL LPS stimulation was given at different times, followed by RT-qPCR to detect the expression of *MHC-I*, *MHC-II*, *CD1C*, *IL6*, *CD80*, and *TLR4*. Data presented as mean ± SEM, *: *p*< 0.05; **: *p* < 0.01.

Following the utilization of *HSPA5*-interfering fragment 183 (*HSPA5*-183) to impede *HSPA5* expression (Figure 9A), HD11 cells were subjected to LPS stimulation. The results obtained at the six-hour mark demonstrated that the *MHC-I* (Figure 9B) and *MHC-II* (Figure 9C) mRNA levels did not change significantly when compared to those observed in the LPS stimulation group. Moreover, *CD80* mRNA levels (Figure 9F) were also inhibited within 6 h after LPS stimulation, whereas *CD1C* mRNA levels (Figure 9D) decreased significantly at 2 h after LPS stimulation. Subsequently, it was observed that IL6 mRNA (Figure 9E) did not undergo a significant alteration within 6 h post-LPS stimulation, and a similar trend was noted for *TLR4* mRNA (Figure 9G). In conclusion, the ability of HD11 to deliver LPS is subject to influence by *HSPA5*. Furthermore, it can be hypothesized that interference with *HSPA5* inhibits LPS recognition through the inhibition of immune-related molecules (*MHC* and cytokines).

## 4. Discussion

Acute heat stress has been demonstrated to result in some detrimental effects on poultry, including decreased feed intake, reduced meat and egg quality, and an elevated mortality rate. These phenomena are likely to cause considerable economic losses to the poultry industry [28,29,30,31,32]. Our findings suggest that the differential expression of genes in chickens subjected to heat stress likely reflects the immune response to such conditions. The majority of these biological processes are associated with immunity, implying that heat stress in the spleen can influence immune function. Notably, only a limited number of genes exhibited differential expression following heat treatment in the bursa of Fabricius, indicating that this organ may be less sensitive to high temperatures than the spleen. Corticosterone is a glucocorticoid hormone secreted by the adrenal cortex and is primarily involved in the body’s stress response. The study conducted by He et al. demonstrated that serum corticosterone levels in chickens increased significantly following exposure to heat stress, a finding that is consistent with the results of the present study [33].

Studies on the role of *HSPA5* (also referred to as *GRP78*) in the immune system showed that *GRP78* regulates inflammation and immune responses through multiple mechanisms [34,35], although such reports are predominantly in mammals. As a major endoplasmic reticulum chaperone molecule, *GRP78* facilitates chemokine and cytokine processing and secretion in mammalian cells [36], as well as being a necessary binding partner for cell-surface *MHC-I* [37]. However, these studies are mostly in mammals, and there have been no studies in chickens. mRNA-seq analysis demonstrated that *HSPA5* plays an important role in immune cell activation after examination of samples from birds under acute heat stress conditions

The majority of *HSPA5* studies in chickens have employed single nucleotide polymorphism (SNP) loci and have focused on the protein’s role in apoptosis. For example, *GRP78* is required for cell proliferation and apoptosis inhibition of embryonic fibroblasts [38], and *BiP*/*GRP78* plays a key role in avian reovirus-mediated apoptosis [39]. However, a rare study examining *GRP78* response to heat stress (35 °C) demonstrated that *GRP78* mRNA levels in chicken heart, liver, brain, and leg muscles first increased (peaking at 3 h) and then decreased after, suggesting that the protein may function in avian immune response as well [40]. This study found that intracellular *HSPA5* overexpression did not directly affect the differential expression of antigen-presenting genes but did accelerate and enhance LPS recognition by HD11. Interference with *HSPA5* could inhibit LPS recognition by HD11 (inhibit *MHC-II* expression). Our work, combined with previous research on avian *HSPA5* heat-stress response, suggests that chicken *HSPA5* may play a role in immune cell activation.

In mammals, some studies have demonstrated that *DUSP1* negatively regulates inflammatory cytokine production during the immune response [41]. *DUSP1* expression, for example, inhibited p38 activation and GATA-3 nuclear translocation, thereby impairing T helper-2 (Th2) cells [42]. Furthermore, *DUSP1* attenuates STAT1 activation through the inhibition of miR155 expression and the induction of SOCS-1 [43], as well as possibly being involved in MAP kinase-independent tumorigenesis [44]. Finally, in *DUSP1*-deficient mice, glucocorticoids induce *DUSP1* expression to inhibit JNK and p38 activation. Therefore, *DUSP1* apparently contributes to the anti-inflammatory effects of glucocorticoids [45,46]. However, none is available on its potential role in immune cell activation of chicken. Similarly to our results on *HSPA5*, we found that *DUSP1* overexpression did not directly affect the differential expression of antigen-presenting genes but did accelerate LPS recognition by HD11, thus serving a key immune function. Interference with *DUSP1* could inhibit LPS recognition by HD11. Although the exact mechanism of *DUSP1’*s role in antigen presentation remains to be investigated, our data strongly suggest that *DUSP1* is important to immune response in chicken.

## 5. Conclusions

In this study, mRNA-seq was conducted on the spleen and bursae of experimental chickens, and it was observed that the expression of *DUSP1* and *HSPA5* in the spleen was significantly diminished in the heat stress group. The results of the cytological experiments demonstrated that the ability of HD11 to deliver LPS is subject to influence by *DUSP1* and *HSPA5*. The inhibition of *HSPA5* and *DUSP1* could suppress the activation of immune cells in chicken immune organs under heat stress. This provides a foundation for further research into the impact of heat stress on immunity.

## Figures and Tables

**Table 1:** The DEGs are associated with immune responses in the spleen and bursa of Fabricius.

Gene Symble	Gene Description	log_2_FC	*p* Value	padj	↑or↓(Up or Down)
Group A					
*FGB*	fibrinogen alpha chain	2.52	7.77 × 10^−6^	0.0066	↑
*FGA*	fibrinogen beta chain	Inf	2.31 × 10^−11^	5.89 × 10^−8^	↑
*FGG*	fibrinogen gamma chain	5.02	7.60 × 10^−10^	1.46 × 10^−6^	↑
*IGSF11*	immunoglobulin superfamily member 11	2.59	7.14 × 10^−8^	9.96 × 10^−5^	↑
*ALDOB*	aldolase B, fructose-bisphosphate	6.11	1.29 × 10^−6^	0.0013	↑
*APOB*	apolipoprotein B (including Ag(x) antigen)	Inf	2.08 × 10^−7^	0.00026	↑
Group B					
*GZMA*	granzyme A (granzyme 1, cytotoxic T-lymphocyte-associated serine esterase 3)	2.12	6.52 × 10^−13^	2.5 × 10^−9^	↑
*DUSP1*	dual specificity phosphatase 1	−1.77	3.63 × 10^−19^	5.59 × 10^−15^	↓
*FGB*	fibrinogen beta chain	−2.29	8.22 × 10^−5^	0.024	↓
*FGA*	fibrinogen alpha chain	#NAME?	1.55 × 10^−10^	3.98 × 10^−7^	↓
*FGG*	fibrinogen gamma chain	−4.40	7.31 × 10^−7^	0.00055	↓
*GC*	group-specific component (vitamin D binding protein)	#NAME?	1.46 × 10^−5^	0.0068	↓
*APOLD1*	apolipoprotein L domain containing 1	−2.80	2.39 × 10^−6^	0.0015	↓
*IGSF11*	immunoglobulin superfamily member 11	−2.49	4.44 × 10^−7^	0.00038	↓
*ALDOB*	aldolase B, fructose-bisphosphate	−6.09	4.13 × 10^−6^	0.0023	↓
*ADAMTS1*	ADAM metallopeptidase with thrombospondin type 1 motif, 1	−1.40	5.84 × 10^−10^	9.97 × 10^−7^	↓
*LOC396380*	glutathione transferase	−1.19	0.000123	0.032	↓
*APOB*	apolipoprotein B (including Ag(x) antigen)	#NAME?	7.37 × 10^−7^	0.00055	↓
*GVIN1*	GTPase, very large interferon inducible 1	1.38	1.81 × 10^−6^	0.0012	↑
*CCL17*	chemokine (C-C motif) ligand 17	−1.39	0.000165	0.039	↓
*HSPA5*	heat shock 70 kDa protein 5 (glucose-regulated protein, 78 kDa)	−0.95	2.76 × 10^−5^	0.011	↓
Group C					
*CD69*	CD69 molecule	3.51	1.9 × 10^−8^	1.06 × 10^−5^	↑
Group D					
*COL21A1*	collagen, type XXI, alpha 1	−1.51	3.25 × 10^−7^	0.00054	↓
*CD69*	CD69 molecule	−2.91	3.88 × 10^−5^	0.034	↓
*HSP90AB1*	heat shock protein 90kDa alpha (cytosolic), class B member 1	−0.71	3.89 × 10^−8^	8.23 × 10^−5^	↓

Note: Group A: the differential genes in the spleen between the control and vaccination groups; Group B: the differential genes in the spleen between the vaccination and heat treatment vaccination groups; Group C: the differential genes in the sac of Fabricius between the control and vaccination groups; Group D: the differential genes in the sac of Fabricius between the vaccination and heat treatment vaccination groups. #NAME? represent no expression in the experimental group; Inf represents no expression in the control group. When log2FC ≥ 1 and *p* val ≤ 0.05, it was up-regulated, and when log2FC ≤ −1 and *p* value ≤ 0.05, it was down-regulated.

**Table 2:** The enriched KEGG pathway of DEGs.

No.	Pathway	*p* Value
Group A		
1	Complement and coagulation cascades	0.001
2	Carbon fixation in photosynthetic organisms	0.018
3	Vitamin digestion and absorption	0.02
4	Fructose and mannose metabolism	0.022
5	Pentose phosphate pathway	0.022
6	Methane metabolism	0.025
7	Fat digestion and absorption	0.031
8	Glycolysis/Gluconeogenesis	0.043
Group B		
1	Ovarian steroidogenesis	0.0004
2	Vitamin digestion and absorption	0.002
3	Glutathione metabolism	0.009
4	Cyanoamino acid metabolism	0.013
5	Complement and coagulation cascades	0.019
6	Taurine and hypotaurine metabolism	0.019
7	Chemokine signaling pathway	0.039
Group D		
1	Synaptic vesicle cycle	0.025
2	Protein digestion and absorption	0.035

Note: The enriched pathways in the table are those with *p* value < 0.05.

**Table 3:** DEGs interaction network of IPA in four alignment groups.

NO.	Network	Genes (Count)
Group A		
1	cellular function and maintenance, hematological system development and function, cellular development	*BCL3*, *BCL*, *CD40*, *↑CD69*, *CD3E*, *IL4*, *EL1B*, *INPP5D*, *MS4A1*, *POU2AF1*, *RIPK2*, *STAT6*, *TGFB1*, *TLR3* (14)
Group B		
1	Nervous system development and function, cancer, cardiovascular system development and function	*FOXO1*, *↑GZMA* (2)
2	cellular movement, hematological system development and function, immune cell trafficking	*CCL4*, *CXCL3*, *↓DUSP1*, *ELF4*, *IL6*, *IL10*, *TGFB2*, *TNF* (8)
Group C		
1	cellular movement, cellular function and maintenance, behavior	*↓CA3*, *CCL15*, *CD59*, *↑CD69*, *CTSG*, *↓DCLK1*, *DCX*, *DRD2*, *GDNF*, *GRIN1*, *HDC*, *LAMC2*, *↑MAP2*, *MAPK1*, *MBP*, *MME*, *↑MMP7*, *MMP8*, *NTF4*, *PP1-C*, *SRC*, *↑TAC1*, *TACR1*, *TFF2*, *TGFA*, *TRPV1*, *↓TUBA1C* (27)
2	Cell-to-cell signaling and interaction, digestive system development and function, hepatic system development and function	*↓FER1L6*, *HLX* (2)
Group D		
1	Cell-to-cell signaling and interaction, cellular growth and proliferation, cellular movement	*ABCB4*, *ACE*, *CALCA*, *↓CD69*, *CNTF*, *CREM*, *CRH*, *CTSG*, *↑ENPP2*, *FCER1A*, *GCG*, *GDNF*, *HDC*, *IL6*, *IL6R*, *MMP8*, *NFAT*, *NR4A1*, *SELE*, *↓TAC1*, *TACR1*, *TFF2*, *TGFA*, *TH*, *TNF*, *TNFRSF11B*, *TNFSF13B*, *TRPV1* (28)

Note: *↑* represents up-regulated of expression, and *↓*represents down-regulated of expression.

**Table 4:** RT-qPCR validation of DEGs.

Gene	Log2(FC)_RNA-seq	Log2(FC)_qPCR
Group A		
*MYL1*	6.852	3.321
*SYT8*	2.386	1.988
*FGB*	2.519	1.769
*ALDOB*	3.027	2.256
*IGSF11*	2.592	2.941
Group B		
*DUSP1*	−1.768	−1.145
*GZMA*	2.118	1.586
*CCL17*	−1.395	−1.16
*FGB*	−2.286	−1.539
*ALDOB*	−6.087	−2.195
*GGT1*	−1.127	−1.041
*HSPA5*	−0.952	−1.233
*IGSF11*	−2.489	−1.615
Group C		
*CD69*	3.513	3.026
*TAC1*	7.934	4.075
*MMP7*	5.248	2.579
*DCLK1*	−2.429	−1.318
*AADAT*	−1.158	−1.502
*MYBPC1*	−3.555	−1.852
Group D		
*CD69*	−2.91	−1.699
*TAC1*	−7.333	−2.133
*ABCB1LB*	1.151	2.018
*COL21A1*	−1.511	−1.092
*ENPP2*	1.291	1.396
*HSP90AB1*	−0.712	−0.845

## Data Availability

The datasets that have been generated during this study are available for acquisition from the corresponding authors.

## Abbreviations

The following abbreviations are used in this manuscript:

DEGs	differentially expressed genes
LPS	lipopolysaccharides
HSPs	heat shock proteins
APCs	antigen-presenting cells
NDV	Newcastle disease vaccine
HI	hemagglutination inhibition

## Appendix A

**Table A1:** The primer pairs information of the related genes.

Gene Symbol	Primer Sequence (5′→3′)	Accession No. in NCBI	Product Size (bp)
*DUSP1*	F: CTGTGCAGAGAGGCCCG	NM_001085359.1	186
	R: GTACAGAGGGGTACCGCAGG		
*GZMA*	F: TGATCAAGCCAAGCTGGGTG	NM_204457.1	149
	R: GGGACAGTAGTCTGGGTAGC		
*CCL17*	F: CCACGATGCTCAGCACAAAG	NM_001293309.1	168
	R: GACAGTCGTGGGAAGTCTCAT		
*FGB*	F: GTGGCCAAATTTTCTGACACCT	NM_001167683.1	195
	R: AAGGACCCTGAGGCTAGAGG		
*ALDOB*	F: AGATCAGTAGCACAACGCCC	NM_001007977.2	101
	R: AATGGGCACCAAGCCATTCT		
*GGT1*	F: TGGGAAAGGCCTTGCATCAG	XM_015275453.1	173
	R: AGGCATCTGCTCCTTCATCG		
*HSPA5*	F: CCTGAGGGGGAGCGCCTGAT	NM_205491.1	111
	R: GGGGTCATTCCAGGTGCGGC		
*IGSF11*	F: ACGCAGCTATCAGACACTGG	XM_004938202.2	130
	R: CTTGGATTCTGCAAAGCGGG		
*MYL1*	F: GAACCCCACAAACGCTGAGA	NM_001044632.1	98
	R: GCATGGGCAGGAACTCTTCAA		
*SYT8*	F: GACTGAGGCTGGAAACAACAC	XM_015286685.1	73
	R: TAGCCTGGCCATTCCTCTGTG		
*AHSG*	F: TTGCAGAACATGCAGTTGAGG	XM_422764.6	74
	R: AGCAAGTACTGAAAACTGTCCAT		
*ABCB1LB*	F: GCAGAAGCAGCGAATAGCAA	NM_204894.2	153
	R: ACTACAACCGTGGTCCGTC		
*COL21A1*	F: ACTGCAACCACTGTCAGTCC	XM_004940463.1	168
	R: GCAATCCTCTTGTCCCACGA		
*CD69*	F: AGAACGCACACCTATGCAGG	XM_015300817.1	124
	R: CGCAACAACCTGGAATTGGG		
*TAC1*	F: ACGGCCAGATCTCTCACAAA	XM_418674.6	125
	R: TCATTTACGCCTTCGCTCGT		
*LECT2*	F: CACGTCGAGAACTGCGACCA	NM_205478.2	113
	R: TTCCCAGCACATACTACAGCC		
*MMP7*	F: GAGAGGGGCTAGGTGGAGAT	NM_001006278.1	132
	R: GGACATTTGAGTGGGCGAGT		
*CD80*	F: AAAGTACCTCCCAAGGCACG	NM_001079739.1	101
	R: ATTTACAGCTGTGGGGTGGG		
*CD86*	F: CCTTGGCCAGGAAAAACACG	NM_001037839.1	116
	R: ACTGCCCCTCATCCACAATC		
*CD1c*	F: CGCGCGTTTTCCAGATCCACAC	NM_001017412.1	186
	R: CGGCCCGGAGGTCAGTGAGAGT		
*MHC-I*	F: ACAAGTACCAGTGCCGCGTG	NM_001097530.1	197
	R: CGCGATGTTGTAGCCCTTCC		
*MHC-II*	F: CCCTCGGCGTTCTTCTTCTAC	NM_001044679.1	128
	R: CCCACGTCGCTGTCGAA		
*IL6*	F: AACGTCGAGTCTCTGTGCTAC	NM_204628.1	171
	R: GAGGATGAGGTGCATGGTGAT		
*IL1B*	F: GCTCTACATGTCGTGTGTGATGAG	NM_204524.2	80
	R: TGTCGATGTCCCGCATGA		
*TLR4*	F: AGTCTGAAATTGCTGAGCTCAAAT	NM_001030693.1	190
	R: GCGACGTTAAGCCATGGAAG		
*GAPDH*	F: GAGGGTAGTGAAGGCTGCTG	NM_204305	113
	R: CATCAAAGGTGGAGGAATGG		

**Table A2:** Interference fragment information of DUSP1 and HSPA5.

Gene	Fragment	Sequence(5′–3′)
*DUSP1*	DUSP1-724	Sense: GCGAUUGACUUCAUAGAUUTT
		Anti-sense: AAUCUAUGAAGUCAAUCGCTT
	DUSP1-1010	Sense: CCACCACAGUCUUUAACUUTT
		Anti-sense: AAGUUAAAGACUGUGGUGGTT
	DUSP1-1909	Sense: CCUCAUAGUUGACUAGAAATT
		Anti-sense: UUUCUAGUCAACUAUGAGGTT
*HSPA5*	HSPA5-183	Sense: GCGUGGAAAUCAUUGCCAATT
		Anti-sense: UUGGCAAUGAUUUCCACGCTT
	HSPA5-412	Sense: GCCACAUAUUCAGGUCGAUTT
		Anti-sense: AUCGACCUGAAUAUGUGGCTT
	HSPA5-985	Sense: GGAUUUCUCUGAGACGCUUTT
		Anti-sense: AAGCGUCUCAGAGAAAUCCTT
*GAPDH*	NC	Sense: UUCUCCGAACGUGUCACGUTT
		Anti-sense: ACGUGACACGUUCGGAGAATT
